# The role of PKA in the translational response to heat stress in *Saccharomyces cerevisiae*

**DOI:** 10.1371/journal.pone.0185416

**Published:** 2017-10-18

**Authors:** Carla E. Barraza, Clara A. Solari, Irina Marcovich, Christopher Kershaw, Fiorella Galello, Silvia Rossi, Mark P. Ashe, Paula Portela

**Affiliations:** 1 Universidad de Buenos Aires. Facultad de Ciencias Exactas y Naturales. Departamento de Química Biológica. Instituto de Química Biológica de la Facultad de Ciencias Exactas y Naturales-Consejo Nacional de Investigaciones Científicas y Técnicas (IQUIBICEN-CONICET). Buenos Aires, Argentina; 2 Instituto de Investigaciones en Ingenieria Genetica y Biologia Molecular "Dr. Hector N. Torres", Buenos Aires, Argentina; 3 The Michael Smith Building, Faculty of Life Sciences, University of Manchester, Manchester, United Kingdom; Medical Faculty Mannheim, University of Heidelberg, GERMANY

## Abstract

Cellular responses to stress stem from a variety of different mechanisms, including translation arrest and relocation of the translationally repressed mRNAs to ribonucleoprotein particles like stress granules (SGs) and processing bodies (PBs). Here, we examine the role of PKA in the *S*. *cerevisiae* heat shock response. Under mild heat stress Tpk3 aggregates and promotes aggregation of eIF4G, Pab1 and eIF4E, whereas severe heat stress leads to the formation of PBs and SGs that contain both Tpk2 and Tpk3 and a larger 48S translation initiation complex. Deletion of *TPK2* or *TPK3* impacts upon the translational response to heat stress of several mRNAs including *CYC1*, *HSP42*, *HSP30* and *ENO2*. *TPK2* deletion leads to a robust translational arrest, an increase in SGs/PBs aggregation and translational hypersensitivity to heat stress, whereas *TPK3* deletion represses SGs/PBs formation, translational arrest and response for the analyzed mRNAs. Therefore, this work provides evidence indicating that Tpk2 and Tpk3 have opposing roles in translational adaptation during heat stress, and highlight how the same signaling pathway can be regulated to generate strikingly distinct physiological outputs.

## Introduction

In response to environmental stress conditions, the cellular proteome is readjusted through signaling pathways that alter different processes connected to transcriptional, translational and post-translational programs.

At suboptimal growth temperatures, *Saccharomyces cerevisiae* activates both transcriptional and physiological protective mechanisms. Genomic expression patterns of *S*. *cerevisiae* have identified several genes involved in carbohydrate metabolism, protein folding and degradation, cytoskeletal reorganization, protein synthesis, ribosome synthesis and processing as differentially regulated upon heat stress [1]; [2]. A genome-wide analysis of the heat shock response has also revealed a positive correlation between the global transcription and translation profiles [3]. It has been demonstrated that heat shock invokes a general translational arrest and mRNA ribonucleoprotein complex aggregation [4]; [5]. Global phosphoproteome studies of *S*. *cerevisiae* have shown that heat stress is associated with extensive changes in protein phosphorylation, highlighting functions in transcription, protein folding and degradation, cell cycle regulation and morphogenesis as particularly pronounced [6].

mRNA localization to mRNP granules is a key event that determines whether a particular mRNA will be translated, silenced or degraded. In *S*. *cerevisiae*, stress induces the formation of mRNA processing bodies (PBs) and stress granules (SGs). Typically, PBs contain translationally repressed mRNA molecules and proteins involved in many functions including mRNA degradation, translation repression, and mRNA quality control. In contrast, the composition of SGs is influenced by the stringency of the particular stress condition that induces their formation [7]; [8]; [4]; [9].

A number of studies in *S*. *cerevisiae* have explored the underlying mechanism of aggregation and composition of PBs and SGs evoked during different severities of thermal stress. For instance, SG formation upon heat stress is independent of translation initiation arrest caused by phosphorylation of eIF2α [10]. It has also been reported that under mild thermal stress conditions (39°C), *S*. *cerevisiae* cells accumulate mostly PBs; while severe heat stress (46°C) induces both PBs and SGs [4]; [5]. SGs evoked by such severe heat stress contain eIF3 subunits, mRNA, eIF4G_2_, Pab1, Ngr1, Pub1, 40S ribosomal subunits and also typical PB proteins such as Dcp2 and Dhh1 [4]; [10]. Although Dhh1, Dcp2, Pub1 and Ngr1 were found associated with SGs evoked by heat stress, these are not required for SG assembly [4]. Following a milder heat shock (42°C), translation elongation factors eEF3 (Yef3) and eEF1Bγ2 (Tef4) and translation termination factors eRF1 (Sup45) and eRF3 (Sup35) accumulate in cytoplasmic foci and provide a platform for assembly of genuine SGs at 46°C. The sequestration of these factors at 42°C would generate alterations in the translation elongation kinetics which could constrain translation initiation. It has also been described that unphosphorylated eIF2α is recruited to dissipate SGs reinforcing the hypothesis that SGs might act as sites where active translation can initiate upon stress relief [10].

The cAMP–PKA pathway plays a major role in the control of metabolism, stress resistance and proliferation in *S*. *cerevisiae*. PKA is a hetero-tetramer composed of two regulatory subunits encoded by *BCY1* gene, and two catalytic subunits encoded by three partially redundant genes, *TPK1*, *TPK2* and *TPK3* [11]. The cAMP–PKA pathway activity is controlled by fermentable sugars and other nutrients as amino acid and phosphate [12]; [13]; [14]. The inactivation of PKA is important during stationary-phase or nutrient starvation [15], and the activation of this pathway is required for a successful cell cycle resumption [16]. It has been documented that the cAMP-PKA pathway plays a role in the transcriptional response to heat stress [17]; [6], but little is understood concerning the potential roles in translational adaptation to thermal stress.

Our previous results indicate that Tpk2 and Tpk3 localize to both SGs and PBs in response to glucose starvation, strong but not mild osmotic stress, and stationary phase [18]; [19], suggesting that the localization of Tpks to SGs/PBs depends not only on the stress type, but also on the stress stringency. Deletion of the *TPK3* or *TPK2* genes also impacts on the capacity of cells to form PBs or SGs, and their ability to arrest translation properly in response to glucose starvation or stationary phase [19]. A protein composition analysis of granules in the *tpk3*Δ strain revealed an inherent similarity to SGs induced by sodium azide, heat shock or ethanol stress [19]; [20]; [4]; [9]. Given that both the process of translational inhibition and the composition of mRNP granules evoked by mild and severe heat stress appear to be different [4], we wondered whether distinct Tpk isoforms might be differentially involved in delineating the cellular response to varying stress stringency.

In keeping with this idea, we find in this work that PKA subunits localize differently depending on the severity of heat stress. After a mild heat stress, Tpk2 re-localizes from the nucleus to the cytoplasm, while Tpk3 localizes to cytoplasmic foci which do not resemble canonical SGs or PBs. In contrast, under severe heat stress conditions, both Tpk2 and Tpk3 associate with SGs. Analysis of PB/SG aggregation, global translation and mRNA translational fitness following severe heat stress suggest that Tpk2 plays a positive role promoting mRNA translation, whereas Tpk3 appears to be involved in translational repression.

## Materials and methods

### Yeast strains, plasmids, media, growth conditions and drug treatments

The strains and plasmids used in this study are listed in S1 Tables. Strains co-expressing Tpk2-GFP or Tpk3-GFP and Rpg1-RFP were constructed essentially as described [21]. The primers used in this study are listed in S1 Tables. The gene-specific cassette containing the C-terminal coding region of *RPG1* fused to *RFP* carrying the natNT2 selectable marker was amplified by PCR using pYM42 as template and the primers Rpg1-RFP F and Rpg1-RFP R. The resulting PCR product was transformed into *TPK2-GFP* and *TPK3-GFP* strains using the lithium acetate method and selected in the presence of 200 mg/ml nourseothricin. Integration at the appropriate *locus* was tested by PCR using the primers: Tif32+183 F and Nat1158 R. The constructed strains were analyzed for positive RFP signal by fluorescence microscopy. Expression of the fusion proteins was tested by immunoblot using anti-Rpg1 antibody. *TPK1*, *TPK2* and *TPK3* genes were deleted using a standard homologous recombination based method [22]. Briefly, a PCR product carrying the *URA3* gene flanked by 50bp of the corresponding deletion target gene was transformed into strains carrying Rpg1-RFP Dcp2-GFP or Rpg1-RFP Pab1-GFP. The primers used for the amplification were: Tpk1-URA For and Tpk1-URA Rev, or Tpk2- URA For and Tpk2-URA Rev, or Tpk3-URA For and Tpk3-URA Rev. Deletions were verified using a standard PCR strategy.

Strains were grown on rich medium containing 2% bactopeptone, 1% yeast extract and 2% glucose (YPD). Synthetic defined medium containing (SD): 0.67% yeast nitrogen base without amino acids, 2% glucose, plus the necessary additions to fulfil auxotrophic requirements was used to maintain the selectable plasmids. Solid media contained 2% agar. Cells were grown until exponential phase at 30°C (OD_600_ 0.4–0.6). Heat shock treatment was performed on exponential cells for 30 minutes at 37°C or 10 minutes at 46°C. For the cycloheximide treatment, cells were grown to exponential phase, treated with 100 μg/ml cycloheximide for 10 minutes at 30°C before heat shock treatment. Cell viability was verified by serial dilution analysis on SD plates, which were incubated for 48 hours at 30°C before photography (S2 Fig).

### Fluorescence microscopy

For fixed-time epifluorescence microscopy, cells were fixed with 3.7% formaldehyde in PBS buffer. For nuclear staining, cells were resuspended in 0.05% Triton X-100 plus 1 μg/ml DAPI (4,6-diamidino-2-phenylindol) for 30 min. 3 μl of cell suspension were applied to poly-lysine-coated glass slides. Confocal images were taken at room temperature by a microscope Olympus FV300 with objective Plan APO 60X, and the images were acquired using Flow View software (FBMC Department, Facultad de Ciencias Exactas y Naturales, Universidad de Buenos Aires, Argentina). The images were processed using Image J (National Institutes of Health) and Adobe Photoshop CS5 software. Representative cells are shown from independent cultures as indicated in each figure. Two categories of localization were distinguished: N>C cells with nuclear fluorescence stronger than cytoplasmic fluorescence (nuclear to cytoplasm ratios of the signal >1.5) and N = C cells with fluorescence evenly distributed over nucleus and cytoplasm (nuclear to cytoplasm ratios of the signal ≤1.5). The data were represented as % of nuclear GFP signal. Granules of approximately 0.8 μm diameter were counted in >100 cells. For clarity the images shown are single Z-sections.

### Crude extracts and immunoblotting

Whole cell lysates for immunoblot analysis with various antibodies were prepared according to a protocol described elsewhere [23]. Proteins resolved by SDS-PAGE were transferred to Protran (GE Healthcare Life Sciences) nitrocellulose membranes. The membrane blots were blocked with 5% non-fat dried milk and incubated overnight with primary antibodies. The antibodies used were: rabbit anti-Rpg1, eIF4E, eIF4G, mouse anti-Pab1 from [8], goat anti-Pyk (Rockland, USA), rabbit anti-GFP was a gift from M. Monte (University of Buenos Aires, Buenos Aires, Argentina), rabbit anti-Rps3 and Rpl35 were gifts from M. Pool (University of Manchester, Manchester, UK). Secondary antibodies used were horseradish peroxidase-conjugated secondary anti rabbit HRP and anti mouse HRP (Sigma).

### Analysis of protein composition in SG enriched fractions

Granule-enriched fractions were prepared as described previously [4]. Heat shock treated wild type cells expressing Tpk2-GFP or Tpk3-GFP and *tpk3*Δ mutant cells expressing Tpk2-GFP were harvested, washed and re-suspended in lysis buffer containing: 50 mM Tris-HCl (pH 7.6), 50 mM NaCl, 5 mM MgCl_2_, 0.1% NP-40, 1 mM β-mercaptoethanol and 1 tablet/10 ml of Complete Mini Protease Inhibitor Mix EDTA-free (Roche). Alternatively, after heat stress and before lysis the cultures were treated with 1% (v/v) formaldehyde during 1 hour on ice-water bath, followed by 0.1 M glycine addition. When indicated, cultures were treated with cycloheximide, as described above, before the heat stress. Cells were lysed by disruption with glass beads at 4°C for 25 minutes. Cell debris was pelleted at 2000 x*g* for 10 minutes at 4°C. An aliquot of the supernatant was used for immunoblot, the rest was centrifuged at 10000 x*g* for 10 minutes at 4°C. Supernatants (cytoplasmic fraction) and pellets (granular fraction) were analyzed by immunoblotting for the presence of translation factors, Tpk2-GFP or Tpk3-GFP and Pyk1 protein.

### Ribosome co-sedimentation analysis

Sucrose density gradients were performed as described previously [24]. Briefly, cells were grown to exponential phase and heat shocked for 10 minutes at 46°C. 10 μg/ml cycloheximide was added and cells were lysed with glass beads. Three A_260_ units of pre-cleared lysate were loaded onto 15–50% linear sucrose gradients. After centrifugation for 2.5 hours at 40.000 rpm using SW41Ti rotor (Beckman), the gradients were fractionated from the top. The A_254_ was measured continuously using an ISCO UA6 gradient collection apparatus. A total of 15 x 0.5 ml fractions were collected across the sucrose gradients. Proteins were precipitated with 10% trichloroacetic acid, washed twice with acetone, resuspended in Laemmli buffer and used for immunoblotting.

### qRT-PCR

RNA was extracted from the collected fractions using a standard Trizol Reagent (Life Technologies, Carlsbad, CA) protocol and resuspended in 20 μl DEPC treated water. 10 ng of a Luciferase mRNA control (Promega) was added to each fraction prior to RNA extraction to control for any influence variable concentrations of RNA might have during the RNA isolation and reverse transcription reactions. Monosomal or polysomal fractions were pooled together. DNA was eliminated using DNAse (Promega). Aliquots of RNA (~10 μg) were reverse-transcribed into single-stranded complementary cDNA using an oligo-dT primer, random primers and M-MLV Taq RT (Promega). For quantitative Reverse Transcriptase (qRT) PCR experiments, primer pairs were designed as presented in S1 Tables. The PCR products were visualized using SYBR Green. Samples were run in triplicate. The proportion of each mRNA within a polysomal fraction was normalised to the Luciferase mRNA control. Once normalised, the total signal for a given mRNA across each fraction of the gradient was determined. Translational activity change was calculated as [(P_fraction_ 46°C/M_fraction_ 46°C)/(P_fraction_ 30°C/M_fraction_ 30°C)] ratio for each mRNA analyzed.

### Kinase assay

Wild type cells expressing GFP-Tpk1-His_6_, GFP-Tpk2-His_6_ or GFP-Tpk3-His_6_ were grown in SD medium at 30°C and then shifted to 46° C during 10 minutes. Purification of His-tagged Tpk was performed as described previously [25]. Protein kinase activity was assayed on purified Tpk. The kinase activity reaction was started by mixing the samples with assay mixture containing 0.15 mM ATP, 1300 dpm.pmol^-1^ and 0.8 mM kemptide [23]. After 30 min at 30°C aliquots were processed according to the phosphocellulose paper method [26]. Protein kinase assays were linear with time and the different protein amounts of each sample.

## Results

### Differential localization of PKA subunits depending on the severity of the heat stress

Regulatory and catalytic subunits of PKA localize to the nucleus, cytoplasm or SGs/PBs depending on a range of external factors including the carbon source, nutrient availability, osmotic conditions or entry into stationary phase [27]; [18]; [19]; [28]. Here we study how PKA subunits localization is affected by moderate and severe thermal stress.

We chose 37° C for 30 minutes and 46° C for 10 minutes as moderate and severe thermal stress respectively based on: a) translational response to severe heat stress (10 minutes at 46°C) was already very well characterized [4], [29], [30]; b) exposure of the cells to 30 minutes at 46°C drastically decreased cell viability (cell viability was verified by a spot assay of serial dilutions on agar plates, S2 Fig), c) availability of transcriptome and mRNA translation correlation data is available in cells submitted to a moderate thermal stress of 30 min at 37°C [3]; an inhibition of translation has been described [31] under this condition as well a greater difference in the localization of Tpk2 and Tpk3 (our own results).

**Fig 1:**
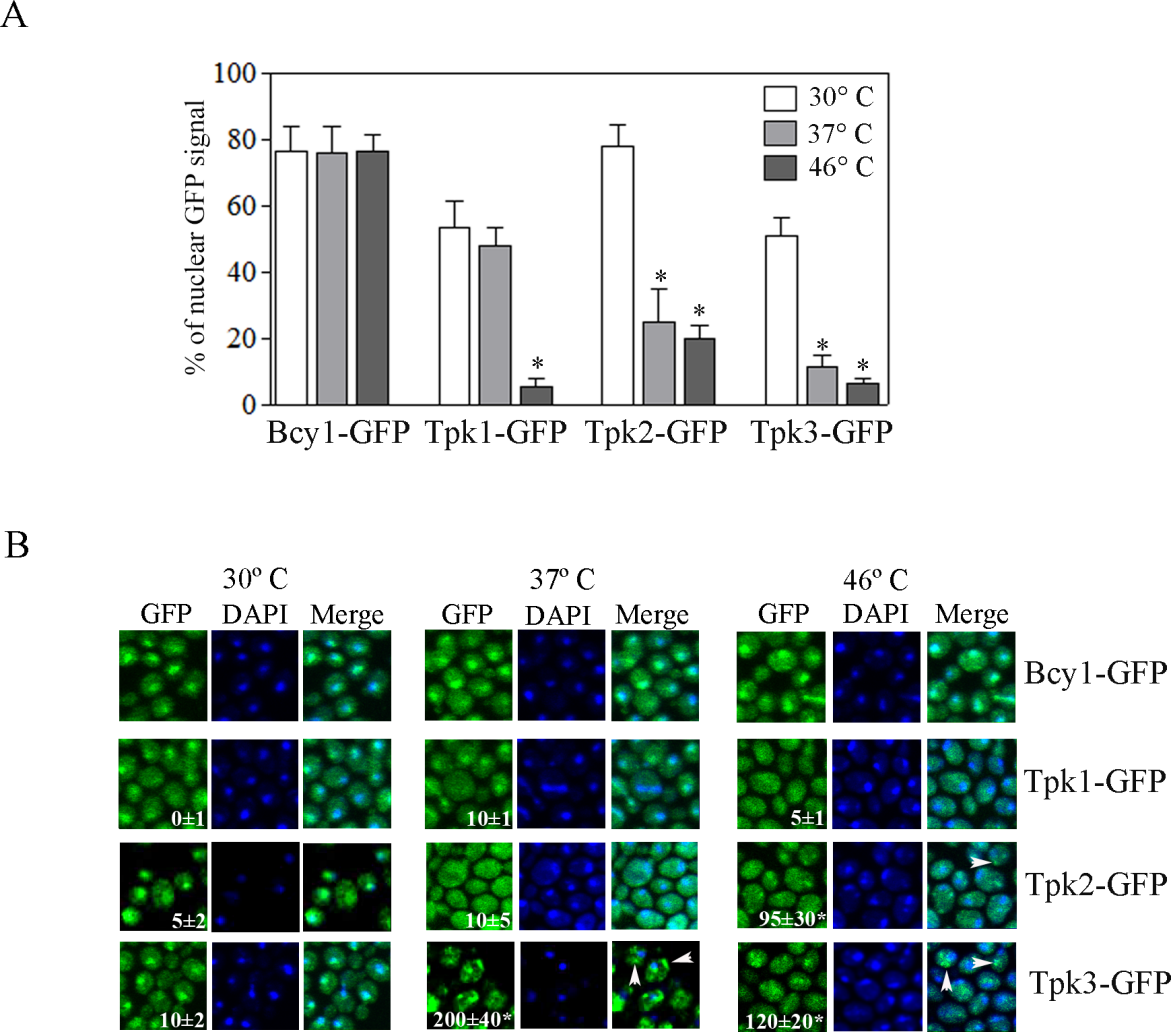
PKA catalytic subunits show a different subcellular localization upon mild and severe heat stress. (A) Subcellular localization of Bcy1-GFP, Tpk1-GFP, Tpk2-GFP or Tpk3-GFP in exponentially growing cells (30°C) and after heat stress at 37°C 30 minutes or 46°C 10 minutes visualized by fluorescence microscopy. Cell nuclei were stained with DAPI. The left graph shows the % of nuclear GFP signal. Values are mean +/- SEM, n = 3. * *p* < 0.05 Tpk1-GFP 30°C *versus* 46°C; Tpk2-GFP 30°C *versus* 37°C and 46°C; Tpk3-GFP 30°C *versus* 37°C and 46°C (ANOVA Bonferroni post-test). (B) The panels show representative images. Numbers inside each photo indicate total granules/100 cells for each of the conditions tested. The arrows show granular localization in the merge channel. Values are mean +/- SEM, n = 3. * *p* < 0.05 Tpk2-GFP 30°C *versus* 46°C; Tpk3-GFP 30°C *versus* 37°C and 46°C (ANOVA-Tukey HSD test).

For this, strains expressing chromosomally tagged Bcy1-GFP, Tpk1-GFP, Tpk2-GFP or Tpk3-GFP were grown to exponential phase in glucose at 30°C and then shifted to 37°C for 30 minutes (mild heat stress) or to 46°C for 10 minutes (severe heat stress). Fig 1A and 1B show that the regulatory subunit, Bcy1-GFP, remained localized mainly in the nucleus under both heat stress conditions (80% nuclear). In contrast, we observed that Tpk1, Tpk2 and Tpk3 catalytic subunits showed different subcellular localization dependent upon the heat stress severity. Tpk1-GFP showed a mixed nuclear-cytoplasmic localization both at 30°C and 37°C (54% nuclear and 48% nuclear respectively). In contrast, after severe heat stress, nuclear Tpk1 levels decreased dramatically (5% nuclear) with a concomitant increase in cytosolic levels (Fig 1A and 1B). Tpk2 also relocated from the nucleus to the cytoplasm when cells were shifted from 30°C (78% nuclear) to 46°C (20% nuclear), although for Tpk2 even a mild heat stress (37°C) treatment was sufficient to trigger the same drastic effect. In addition, while as a response to mild heat stress Tpk2-GFP exhibited a homogeneous cytoplasmic distribution, after exposure to 46°C, Tpk2-GFP localized to cytoplasmic foci (Fig 1A and 1B). Tpk3-GFP also localized to cytoplasmic foci upon both mild and severe heat stress. Indeed, mild stress elicited a higher number of Tpk3-GFP granules per cell than severe heat stress (Fig 1A and 1B). The alterations in localization were not linked to changes in overall expression levels of Tpk1-GFP, Tpk2-GFP and Tpk3-GFP, as levels of protein were unchanged in response to the different severities of thermal stress (S1A Fig).

These results suggest that the subcellular localization of the catalytic subunits of PKA is differentially regulated in response to changing temperature conditions, and that for Tpk2 and Tpk3 in particular, the degree of accumulation in cytoplasmic foci is dependent on the strength of the heat stress.

### Tpk2 and Tpk3 associate with PBs and SGs under heat stress

**Fig 2:**
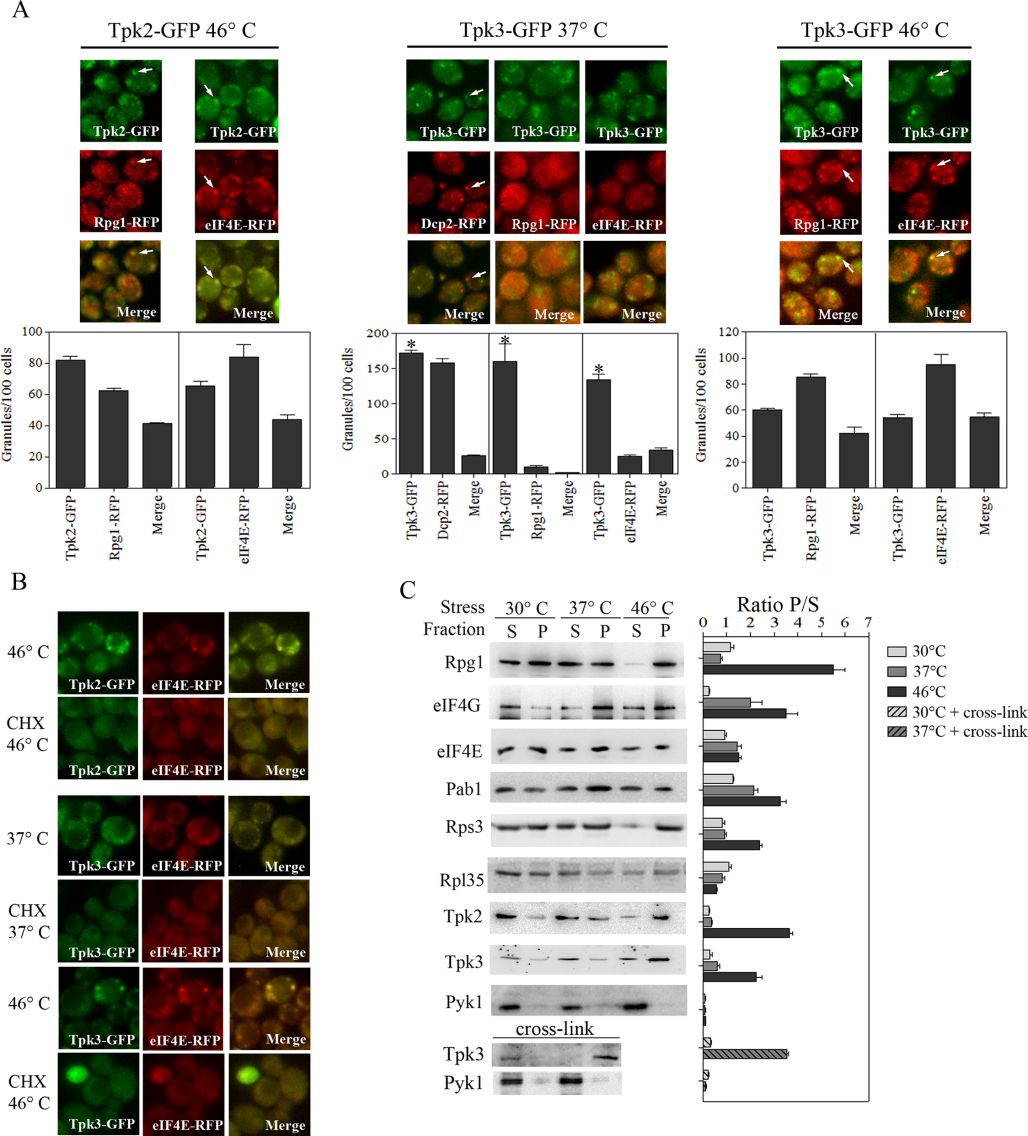
Characterization of Tpk2 and Tpk3 granules evoked during mild and severe thermal stress. (A) Cells co-expressing Tpk2-GFP or Tpk3-GFP and Rpg1-RFP or eIF4E-RFP and Tpk3-GFP and Dcp2-RFP were grown to exponential phase (30°C) and then incubated at 46°C for 10 minutes or 37°C for 30 minutes. Co-localization was determined by confocal microscopy. Arrows indicate Tpk-GFP granular localization and merge. Lower graphs show the quantitation of Tpks, Dcp2, Rpg1, eIF4E and merge granules/100 cells under each thermal stress condition. Values are mean ± SEM, n = 3. * *p* < 0.05 Tpk3-GFP *versus* Tpk3-GFP/Dcp2-RFP merge; Tpk3-GFP *versus* Tpk3-GFP/Rpg1-RFP merge; Tpk3-GFP *versus* Tpk3-GFP/eIF4E-RFP merge at 37°C (ANOVA-Tukey HSD test). (B) Effect of cycloheximide on the Tpk2 and Tpk3 assembly on heat stress evoked SGs. Cells co-expressing Tpk2-GFP or Tpk3-GFP and eIF4E-RFP were pre incubated or not with cycloheximide 100 μg/ml for 10 minutes before heat stress (CHX). Tpk2-GFP and Tpk3-GFP granule formation was analyzed as described in A. (C) Biochemical analysis of Tpk2 and Tpk3 granules evoked by heat stress. Wild type cells expressing Tpk2-GFP or Tpk3-GFP were grown to exponential phase in YPD and subsequently incubated at 30°C, 37°C for 30 minutes or 46°C for 10 minutes. When indicated, cells expressing Tpk3-GFP incubated at 30°C or 37°C 30 minutes were subsequently cross-linked by treatment with 1% (v/v) formaldehyde. Representative blots are shown. The results for the translation markers in Tpk2-GFP cells or Tpk3-GFP cells were similar. Right graph shows the ratio P/S of the abundance of each protein determined by densitometric quantification of the bands. Values are mean ± SEM, n = 2.

To further characterize the Tpk3 and Tpk2 foci observed after heat stress, we performed co-localization experiments with PB and SG marker proteins (Fig 2A). We used strains expressing Tpk2-GFP or Tpk3-GFP; and Dcp2-RFP (as a marker for PBs), or eIF4E-RFP, or Rpg1-RFP (as markers for SGs). Cells were subjected to mild (37°C) or severe (46°C) heat stress and subcellular co-localization was analyzed.

For the Tpk2-GFP and Tpk3-GFP granules that were induced by severe heat stress a partial overlap was observed with the markers for SGs (40–50% overlap) (Fig 2A). However, for Tpk3-GFP granules induced by mild thermal stress, virtually no overlap was observed with Dcp2, eIF4E or Rpg1. Therefore, it seems unlikely that the Tpk3-GFP granules formed under these conditions are either PBs or SGs. To further evaluate the connection between these Tpk3-GFP granules induced by mild heat stress and translational control, we tested whether the aggregation of Tpk3 observed at 37°C is dependent on translation inhibition (Fig 2B). A pretreatment of cells with cycloheximide causes polyribosome stalling on mRNAs preventing the formation of both SGs and PBs in response to stress [32]; [7]; [4]. Similar to the SGs evoked by heat stress, no Tpk3 foci were observed in mild heat stressed cells pre-treated with cycloheximide, suggesting that Tpk3 accumulation in foci is due to the inhibition of translation in response to the heat stress. As previously described [31], our experimental mild stress conditions 30 minutes at 37°C inhibits translation.

Equally, the Tpk2 and Tpk3 granules observed following severe heat stress did not form if the cells were pretreated with cycloheximide, consistent with the partial overlap with SG components described above.

In order to explore the composition of granules induced by the different severities of heat stress, we performed a biochemical analysis on granule-enriched fractions obtained after a differential centrifugation of unstressed or heat-stressed cells expressing Tpk2-GFP or Tpk3-GFP (Fig 2C). The overall levels of a number of translation initiation factors, as well as Tpk2 and Tpk3 were unchanged after the heat stress treatment (S1B Fig). As pre-treatment with cycloheximide prevents formation of SGs evoked by heat stress (Fig 2B), cycloheximide pre-treatment also prevented the enrichment of translation factors into the granule-enriched fraction (S1C Fig). Granules from cells exposed to moderate thermal stress were enriched for eIF4G, Pab1 and, to a lesser extent eIF4E (bars 30°C *versus* 37°C). No enrichment of eIF3 subunits (Rpg1), ribosomal subunits (Rps3 and Rpl35) or PKA catalytic subunits (Tpk2 and Tpk3) was observed. Pyk1 (a glycolytic enzyme) was used as a control for the absence of cytoplasmic contamination in the pellet fractions and, as expected, was only detected in the supernatant fractions. Therefore, as judged from the composition of the granules produced under mild thermal stress conditions, it appears that the translational arrest is not leading to aggregates that carry the ribosomal subunits or eIF3 complex. This is similar to what has been observed for glucose starvation, where eIF4E, eIF4G, Pab1 and certain RNA binding proteins, but not eIF3 or the ribosomal subunits, form EGP-bodies as a form of SGs in a kinetically distinct manner to the production of PBs [8].

It is noteworthy that the degree of enrichment for the Tpk3 protein in granule fractions is higher under severe heat stress than under mild conditions (Fig 2C bars 46°C *versus* 37°C), whereas from the fluorescence microscopy it can be observed that the Tpk3 granules were similar regardless of the severity of thermal stress (Fig 1B). One possible explanation is that the Tpk3 granules formed at 37°C are labile and a proportion does not survive the fractionation procedure. Therefore, to stabilize interactions between Tpk3 and mRNP granules, we performed formaldehyde crosslinking treatment post heat stress. As shown in Fig 2C, an enrichment of Tpk3 in the granule fraction was observed under these conditions. This result suggests that the nature of the Tpk3 interaction with mRNP granules is quite labile under mild heat stress conditions.

Consistent with the microscopy and the cycloheximide results presented above, the biochemical analysis of granule fractions after severe heat stress indicates that eIF4E, eIF4G, Pab1, Rpg1 and Rps3 are enriched, whereas Rpl35 is not present in the granular fraction after 46°C treatment. These results are in agreement with previous descriptions of robust heat shock promoting the formation of yeast SGs containing both eIF3 and the 40S ribosomes [4]. Moreover, we observed that both Tpk3 and Tpk2 co-fractioned with these severe heat stress granules (Fig 2C). Overall, the results from this section indicate that Tpk2 and Tpk3 localize to SGs evoked by severe heat stress, but following mild heat stress only Tpk3 localizes in a more labile manner to granules harboring the eIF4G, Pab1p and eIF4E translation initiation factors.

**Fig 3:**
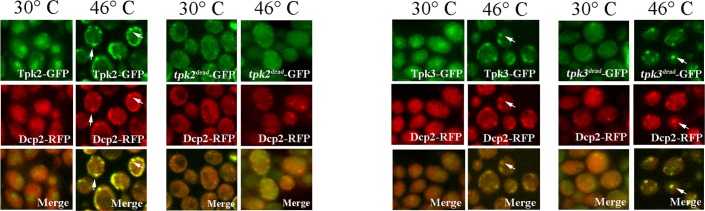
Characterization of kinase dead *tpk2* and *tpk3* granule localization evoked by severe heat stress. Cells co-expressing Tpk2-GFP, *tpk2*^*dead*^-GFP, Tpk3-GFP or *tpk3*^*dead*^-GFP and Dcp2-RFP were subjected to severe heat stress as described in Fig 2A. Co-localization was determined by confocal microscopy. Arrows indicate Tpk-GFP granular localization and merge.

To further characterize the mechanism by which Tpk2 and Tpk3 localize into heat stress evoked foci, we performed experiments employing strains expressing kinase dead *tpk2* (*tpk2*^*dead*^-GFP) or kinase dead *tpk3* (*tpk3*^*dead*^-GFP) and Dcp2-RFP (as PB marker) (Fig 3). Mutants and wild type cells were subjected or not to severe heat stress. After severe heat stress, kinase dead *tpk2*-GFP was not accumulated, indicating that Tpk2 activity is required for its association with Dcp2 granule evoked by severe heat stress. The strain carrying Tpk3 kinase dead version showed significant aggregation of Dcp2-RFP even at optimal temperature, similar to that observed in *tpk3*Δ strain [19]. Dcp2 granule localization of mutant *tpk3*^*dead*^-GFP increased in response to severe heat stress, showing that Tpk3 activity is not required for its accumulation in response to heat stress.

To directly examine Tpks kinase activity under the unstressed and severe heat stress conditions, we performed an *in vitro* PKA kinase assay (S1G Fig). The kinase activity of each purified Tpk was similar pre and post heat stress. These results suggest that heat stress did not affect intrinsic Tpk catalytic activity.

### Tpk2 and Tpk3 play different roles in SGs and PBs aggregation in response to severe heat stress

**Fig 4:**
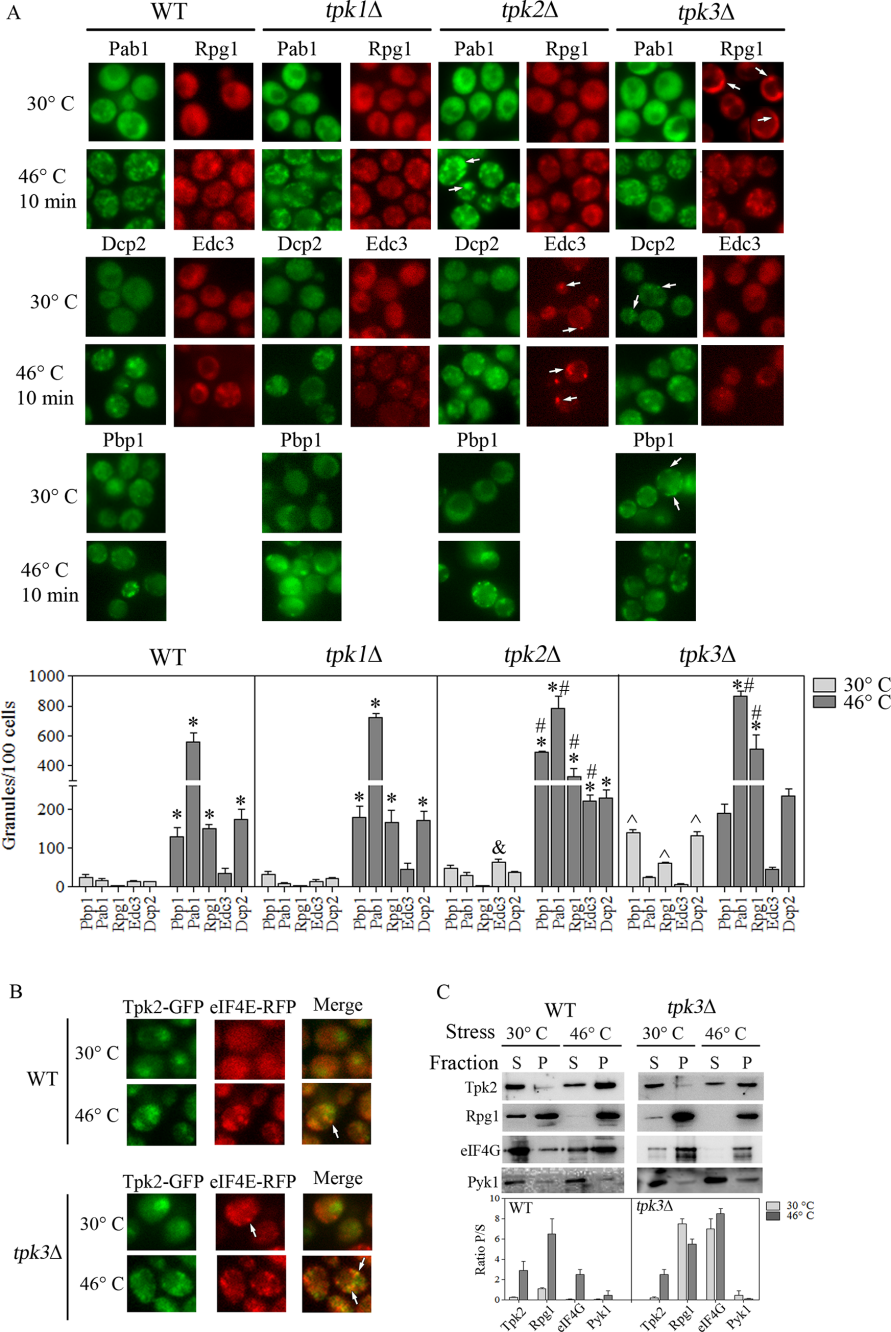
PKA catalytic subunits differentially affect the SGs and PBs aggregation in response to severe heat stress. (A) Wild type (WT), *tpk1*Δ, *tpk2*Δ and *tpk3*Δ expressing Pbp1-GFP, Edc3-RFP, Dcp2-RFP, Pab1-GFP or Rpg1-RFP were grown to exponential phase in YPD (30°C) and incubated at 46°C during 10 minutes. PBs and SGs aggregation were analyzed by fluorescence microscopy. The arrows show granular localization. The graph shows the amount of granules/100 cells. Bars represent the mean ± SEM, n = 3. * *p* < 0.05, 30°C *versus* 46°C; # *p* < 0.05, *tpk2*Δ 46°C or *tpk3*Δ 46°C *versus* WT 46°C; & *p* < 0.05, Edc3 *tpk2*Δ *versus* Edc3 WT 30°C; ^ *p* < 0.05, Pbp1, Rpg1, Dcp2 *tpk3*Δ *versus* Pbp1, Rpg1, Dcp2 WT 30°C (ANOVA Bonferroni post-test). *tpk3*Δ Rpg1-RFP panel results from a montage of images. (B) WT and *tpk3*Δ mutant cells expressing Tpk2-GFP and eIF4E-RFP were incubated at 46°C for 10 minutes. Tpk2-GFP and eIF4E-RFP co-localization was analyzed by confocal microscopy. (C) Tpk2-GFP enrichment in granular fractions was analyzed as described in Fig 2. Representative blots are shown. The graph shows the ratio P/S of the abundance of each protein determined by densitometric quantification of the bands. Values are mean ± SEM, n = 2.

Previously it has been described that severe heat stress resulted in the formation of foci containing: mRNA, Rpg1, eIF4G_2_, Pab1, Ngr1, Pub1, 40S ribosomal subunits and also typical PBs protein such as Dcp2 [4]. However, an intermediate level of heat stress induced the aggregation of the mRNA decapping enhancer and PB marker Edc3 [5]. Since Tpk2 and particularly Tpk3 activity are required for the dynamic assembly of PBs and SGs in response to glucose starvation [19], we evaluated the role of each PKA catalytic subunit in the localization of a number of translation and mRNA decay factors. To this end, we constructed strains deleted for each *TPK* gene that express proteins marking SGs (Pbp1-GFP, Pab1-GFP and Rpg1-RFP) and PBs (Edc3-RFP and Dcp2-GFP). In wild type cells exposed to severe heat stress, Pbp1, Pab1, Rpg1, and Dcp2 all showed significant localization to granules in terms of the number of granules observed per 100 cells (Fig 4A, WT panels 30°C *versus* 46°C). Granules were observed for Edc3, but the difference with unstressed cells was not significant. The *tpk1*Δ mutant strain exhibited, after heat stress, a profile of protein aggregation similar to the one of the wild type strain (Fig 4A, *tpk1*Δ panels).

For the *tpk2*Δ strain, after severe heat stress an even higher degree of aggregation was observed for the SG and PB marker proteins compared to wild type (Fig 4A, *tpk2*Δ panels). In addition, in this strain the Pab1 granules in particular are larger when compared to those observed in wild type cells as verified by size measurements of Pab1-GFP granules (S1F Fig). Furthermore, a significant number of Edc3 containing granules were observed even in unstressed exponential growth conditions. These results suggest that Tpk2 activity negatively regulates SGs/PBs aggregation in cells.

The *tpk3*Δ mutant cells also exhibited changes in SGs/PBs aggregation. In keeping with our previous results [19], exponentially growing *tpk3*Δ mutant cells exhibited a high number of Dcp2 containing granules. Also in unstressed conditions, we observed a small increase in the basal level of Rpg1 and Pbp1 containing granules, while Pab1 and Edc3 showed a diffuse cytoplasmic distribution (Fig 4A, *tpk3*Δ 30°C panels). After severe heat stress, the number of Pab1 and Rpg1 containing granules increased in comparison with similarly stressed WT cells, while Pbp1 and Dcp2 containing granules remained unchanged. As in WT cells, Edc3 remained diffusely distributed in the cytoplasm in stressed *tpk3*Δ mutant cells (Fig 4A, *tpk3*Δ 30°C *versus* 46°C), in agreement with previous studies that indicate that Edc3 as well as Lsm4 are not required for Dcp2 accumulation upon severe heat shock [4].

An intriguing consideration is whether the absence of Tpk3 allows other granule associated catalytic subunits of PKA, such as Tpk2, to become more heavily associated with any mRNP granules. Therefore, while the constitutive translation initiation factor containing granules observed under non-stress conditions in the *tpk3*Δ strain do not contain Tpk2, this PKA subunit does accumulate in the granules observed after severe heat stress in a similar manner to the wild type strain (Fig 4B). These changes in the subcellular distribution of Tpk2-GFP and translation factors in a *tpk3*Δ background upon heat shock were further evaluated in granule-enriched fractions. In contrast to WT cells and in agreement with the microscopy results (Fig 4A and 4B), the granule-enriched fraction from unstressed *tpk3*Δ cells shows a high level of translation factors, such as Rpg1 and eIF4G (Fig 4C). After heat stress Tpk2-GFP was found in the granule fraction for both WT and *tpk3*Δ strains, as expected from the microscopy results (Fig 4B).

Overall, these results suggest that the *TPK3* gene deletion promotes the formation of abnormal granules that contain several translation factors under non-stressed conditions, and that this granule is remodeled in response to heat stress acquiring Tpk2.

### Tpk2 and Tpk3 have different roles in translational regulation following severe heat stress

**Fig 5:**
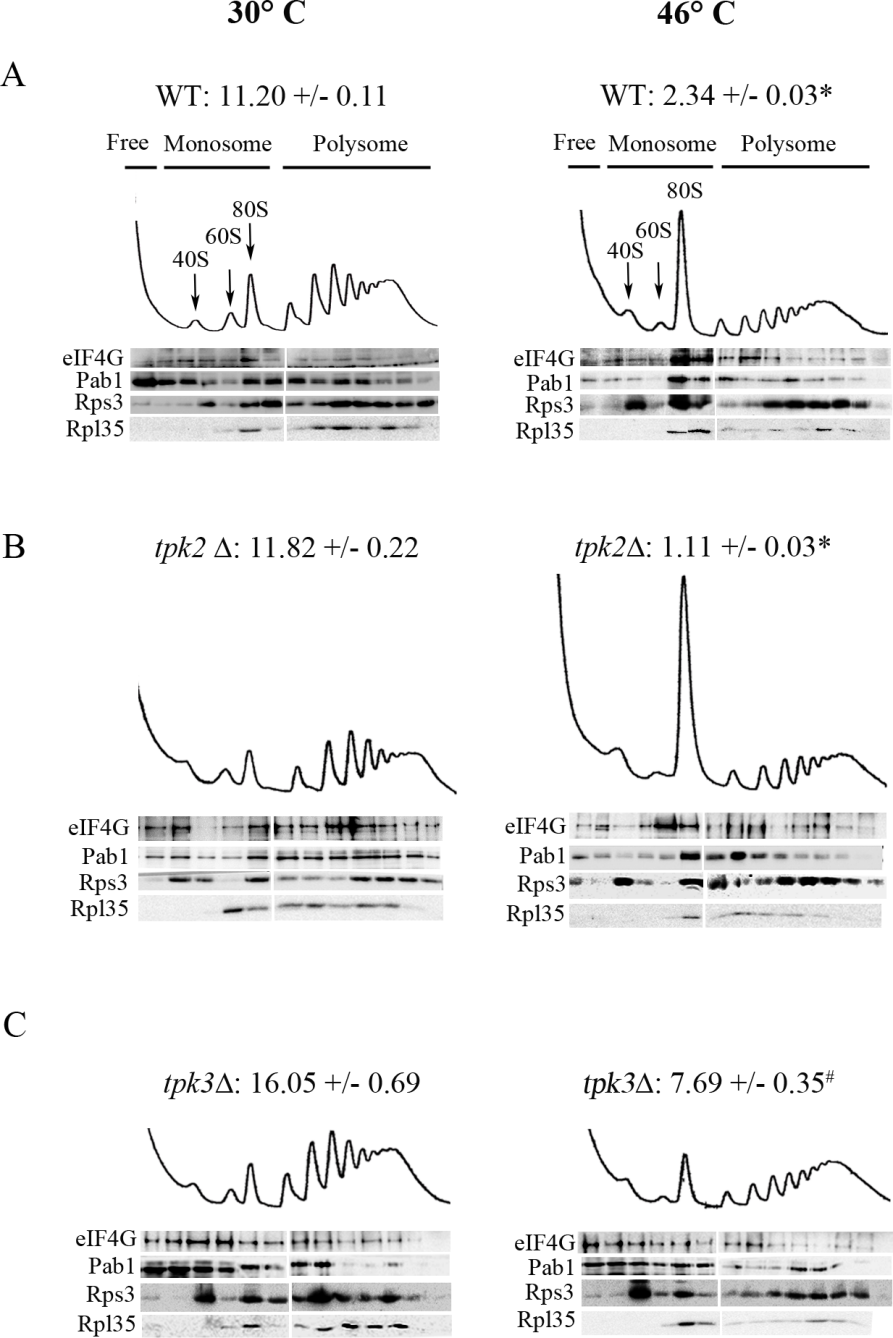
Tpk2 and Tpk3 show an opposite role in translational arrest in response to severe heat stress. Polysomal profile analysis and immunoblots of 15–50% sucrose gradient fractions from WT (A), *tpk2*Δ and *tpk3*Δ cells grown to exponential phase in YPD (30°C) and subjected to severe heat stress (46°C for 10 minutes). Free, monosome and polysome regions are indicated over the polysome profile. The numbers represent the polysome/monosome area ratio (mean +/- SEM, n = 3). * *p* < 0.05 WT and *tpk2*Δ 30°C *versus* 46°C; # *p* < 0.05 *tpk3*Δ *versus* WT and *tpk2*Δ 46°C (ANOVA Bonferroni post-test). Quantification of translation factors in monosome fraction (M) and polysome fraction (P) are shown in S1E Fig.

As described above, deletion of *TPK2* or *TPK3* genes differentially affects the aggregation of several translation factors upon severe heat stress. To analyze the role of Tpk1, Tpk2 and Tpk3 in translational regulation, we studied the distribution of translation factors across polysome profiles in the *tpk1*Δ, *tpk2*Δ and *tpk3*Δ mutant strains. Deletion of *TPK1*, *TPK2* or *TPK3* individually did not affect translation factor protein levels (S1B Fig). As expected from previous studies, wild type cells subjected to severe heat stress exhibited an increase in the 80S monosome peak with a concomitant decrease in polysomes indicative of an inhibition of translation initiation [4] (Fig 5A). The translation factors eIF4G, Pab1, Rps3 and Rpl35 were detected in polysomal fractions in unstressed conditions (30°C), as described previously [33]. For the translation factors associated with mRNA selection to be translated, eIF4G and Pab1, the polysome association likely reflects initiation events occurring on mRNAs that are already being translated by multiple ribosomes. After heat stress, ribosomal proteins Rps3 and Rpl35, as well as eIF4G and Pab1 relocate to the monosomal and submonosomal fractions (Fig 5A, S1E Fig).

Similar polysomal profile and fractional distribution of translational factors between t*pk1*Δ mutant strain and wild type cells were observed (S1D and S1E Fig). The polysomal profile obtained from wild type cells was similar to those presented in Fig 5.

For the *tpk2*Δ mutant strain in unstressed condition, the polysome profile was similar to that observed in wild type cells, while under heat stressed conditions the change in polysome to monosome ratio was more pronounced in the *tpk2*Δ mutant than in wild type cells. No major differences in fractional distribution of translation factors were observed (Fig 5B, S1E Fig).

Deletion of *TPK3* drastically altered the response to stress in terms of translational activity and sedimentation patterns of the analyzed translational factors in a way opposite to *TPK2* (Figs 5C and S1E). Under normal growth conditions, *tpk3*Δ strain showed a marked increase in the eIF4G and Pab1 levels associated with submonosomal fractions in comparison with wild type cells. Most strikingly, the *tpk3*Δ mutant strain exhibited a weaken polysome/monosome area ratio reduction in response to heat stress. The pattern of translation factor fractionation across the polysome profile was almost unchanged in response to severe heat stress. Therefore, in the *tpk3*Δ mutant following severe heat stress, there was an impaired translation arrest and, possibly as a result, a lack of translation factors re-localization. Similar to the result for *tpk3*Δ, deficiencies in PKA activity have previously been shown to dramatically reduce the level of inhibition of translation observed after other stresses, such as glucose depletion [24]. However, the deficient mutant phenotype used were considerably more severe than those used here. Indeed, when single *TPK1*, *TPK2* or *TPK3* gene deletion strains were tested under glucose depletion conditions, little or no deficiency in the translational inhibitory response was observed [19].

**Fig 6:**
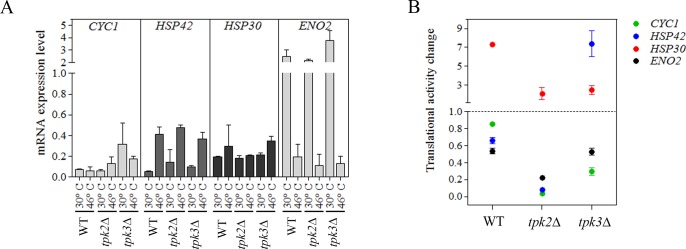
Tpk2 and Tpk3 affect the translational response to severe heat stress. (A) mRNA expression level was determined by q-RT-PCR on samples before and after severe heat stress. The value represents mean +/- SEM, n = 2. *ACT1* mRNA was used as a control. (B) The RNA collected from sucrose gradient fractions was pooled into monosome (M) and polysome (P) fractions. The mRNA distribution was analyzed by qRT-PCR and quantified relative to a luciferase mRNA control. Translational activity change was calculated as described in Materials and Methods. The values represent mean from two independent samples. The value represents mean +/- SEM, n = 2.

Given the differences observed at the global translational level in the *TPK2* and *TPK3* mutants, we chose to delve deeper into the question as to whether the translational response to severe heat stress was altered for a range of specific mRNAs that respond differently in terms of their translational response to heat shock. We chose to study the *ENO2*, *CYC1*, *HSP30* and *HSP42* genes, as after heat stress, these genes/mRNAs are altered either in their abundance (transcription rate and mRNA stabilization) or at the translational level [1], [34]; [3]; [6]. The level of these selected mRNAs was assessed using qRT-PCR in wild type, *tpk2*Δ and *tpk3*Δ mutant cells pre and post heat stress (Fig 6A).

*ACT1* mRNA levels were used as a control to normalize the mRNA targets, as this mRNA is not altered by thermal stress [35, 36]. In wild type cells subjected to heat stress, each mRNA showed a different expression profile. Upon severe heat stress, the level of *CYC1* and *HSP30* mRNAs remained relatively unchanged, *HSP42* mRNA was heavily induced and *ENO2* mRNA levels were reduced dramatically. As described previously [35], deletion of either the *TPK2* or *TPK3* genes did not affect significantly the mRNA levels of the analyzed genes in unstressed conditions (Fig 6A). Furthermore, both *tpk*2Δ and *tpk3*Δ mutant cells harbored similar levels of mRNA compared to wild type cells after severe heat stress, suggesting that the Tpk2 and Tpk3 catalytic subunits of PKA do not impact upon the transcript abundance via regulation at the level of transcription or mRNA stability for this set of analyzed genes/mRNAs.

However, the global polysome profiles and microscopy described above suggest that Tpk2 or Tpk3 PKA catalytic subunits might also impact upon specific mRNA fate at the level of translation and/or localization. To evaluate the translational response for *ENO2*, *CYC1*, *HSP30* and *HSP42* mRNAs, their levels were determined from fractions across a polysomal gradient. More specifically, gradient sedimentation was used to separate polysomal and monosomal associated mRNAs before and after severe heat stress and the level of the selected mRNAs were determined using qRT-PCR. The abundance of each mRNA was measured in polysome and monosome fractions to give an assessment of the translation state: the ratio of mRNA level in polysomes to mRNA level in monosomes. This ratio of translation states of stressed and unstressed cells was taken as a measure of the change in translation activity and defined so in Fig 6B [33], which depicts this change for each of the mRNAs analyzed. Wild type cells showed a reduction in the translational activity of both *ENO2* and *HSP42* mRNA, while *CYC1* mRNA was less responsive. On the contrary, *HSP30* mRNA translational activity was dramatically increased following severe heat stress. These data parallel what happens for *HSP30* and other heat shock protein mRNAs after nutrient starvation where translation becomes activated following glucose starvation [37]. Therefore, it appears that *HSP30* mRNA is an example where translation is upregulated to facilitate adaptation to stress, even though global translation is repressed. Deletion of the *TPK2* gene promoted a severe reduction in the translational activity change in response to heat stress for all of the transcripts analyzed, although the general trend shown by wild type cells was retained (Fig 6B). It is possible that this reduction reflects the greater level of inhibition that is observed in the global polysome profiles in this null strain (Fig 4B). In contrast, the *tpk3*Δ mutant strain showed distinct and varied alterations in the translational activity in response to severe heat stress. With the exception of *ENO2* mRNA, all of the other selected mRNAs exhibited changes. For instance, the *HSP42* mRNA showed a very high and positive change in translational activity relative to the wild type. In contrast, for the *HSP30* mRNA, less change in the translation activity was observed in the *TPK3* mutant relative to the wild type.

Thus, it appears that the Tpk2 and Tpk3 isoforms differentially affect translational activity globally as well as at the level of specific mRNAs, where several different alterations to translational activity were observed.

## Discussion

### Differential PKA catalytic subunit localization as a response to heat stress stringency

Different PKA subunits have been shown to display diverse subcellular localizations depending on the quality of the carbon source, level of glucose, degree of osmotic stress and in stationary phase [27]; [18]; [28]. Moreover, we have shown previously that two of the catalytic subunits of PKA, Tpk2 and Tpk3, accumulate in PBs and SGs in response to glucose starvation and in stationary phase [18]; [19]. In this study, we demonstrate that the subcellular localization of the catalytic subunits and the regulatory subunit of PKA is differentially controlled by thermal stress. Tpk1 re-localizes from the nucleus to the cytoplasm after severe heat stress, whereas Tpk2 and Tpk3 subunits show different granular localization patterns according to the severity of the thermal stress. Our observations contribute to the concept that different severities of the same stress induce distinct cellular responses, and highlight a potential role for different isoforms of PKA in these distinctions.

The granular localization of Tpk2 and Tpk3, evoked by heat stress, was not observed if cells were pre-treated with cycloheximide. This indicates that, similar to PBs and SGs, the Tpk3 foci induced by mild heat stress, and the Tpk2 and Tpk3 foci evoked by severe heat stress are dependent on the repression of translation initiation. This raises the question as to the nature of the relationship between these Tpk foci and PBs/SGs. SGs, in particular, vary compositionally according to the stress that causes their accumulation. For instance, glucose starvation causes the formation of EGP-bodies, a form of yeast stress granules that contain eIF4E, Pab1 and eIF4G as well as some RNA binding proteins; whereas, severe heat stress and ethanol stress have been characterized as being correlated with the formation of stress granules harboring these same factors together with eIF3 and the 40S ribosomal subunit. So while EGP-bodies appear to resemble the mRNA granules that are induced by ischemia in neurons [38], the yeast stress granules formed upon severe heat stress are more akin to classical mammalian stress granules [39].

In this study, upon mild heat stress eIF4G, Pab1 and eIF4E were enriched in granular fractions. This suggests that mild heat stress could exert a translational arrest resembling the one evoked by glucose depletion and leading to a mRNP granule comparable to an EGP body [8]. In response to severe heat stress, granule enriched fractions did not contain Rpl35 -a component of 60S ribosomal subunit- but contained translation factors from the pre-initiation complex including eIF3 and the 40S ribosomal subunit. These results support previous reports that demonstrate that the translational arrest after a severe heat stress occurs at the 48S complex level [4]. We here describe for the first time, that Tpk2 and Tpk3 colocalize with SGs induced by severe heat stress. This adds to a growing list of connections that have been discovered between signaling pathways and SGs [39].

In response to heat stress, localization of Tpk2 was dependent on its catalytic activity while the one of Tpk3 was not, suggesting that each catalytic subunit might have different mechanisms of aggregation in response to severe heat stress. This would be different to the mode of aggregation in response to glucose starvation [19], indicating that the same catalytic subunits could be subjected to different aggregation mechanisms depending on the stress conditions.

A range of different signaling molecules, such as protein kinases, phosphatases, GTPases and ubiquitin modifying enzymes localize to SGs. For instance, components of the TORC1 complex are sequestered to SGs upon heat stress, controlling TORC1 reactivation during recovery of cells from heat stress [40]. Under certain stress conditions, the multi-functional adaptor RACK1 is sequestered in SGs in order to prevent p38/JNK1 signaling and the activation of apoptosis [41]. Similarly, activated RhoA GTPase and its downstream kinase ROCK1, which signal JNK-mediated apoptosis, are sequestered into stress granules to prevent this process [42]. Thus, the punctate localization of Tpk2 and Tpk3 in cells exposed to 37°C or 46°C could represent a mean to sequester specific isoforms of the kinase such that cytosolic activity of some isoforms is limited, while others are active. Alternatively, given recent evidence on the role of liquid phase transition in signaling [43] and cytosolic RNP granule formation [44], it is possible that SGs might represent regions where the activity of enzymes or processes are concentrated. Hence, another overlapping possibility to sequestration is that the activity of specific isoforms of PKA might be restricted to targets within the granules.

### Tpk2 and Tpk3 role in translational response evoked by thermal stress

We have already reported that *TPK3* or *TPK2* deletions affect the capacity of cells to form granules and inhibit translation in response to glucose starvation or in stationary phase [19]. Here we have found that the deletion of *TPK2* or *TPK3*—but not *TPK1*- had differential consequencies on the formation of SGs/PBs evoked by thermal stress. Deletion of the *TPK2* gene caused an increase in the number and size of SGs and PBs in comparison to wild type cells upon severe heat stress. In addition, the level of translation inhibition in response to heat stress was increased when compared to wild type cells, both at the global and mRNA-specific level. These results suggest that Tpk2 catalytic subunit seems to have a negative role on translational inhibition, SGs/PBs formation and mRNA translation in response to heat stress.

It has been described that granule-enriched fractions from *tpk3*Δ mutant cells contain Rpg1, Rps3, Dcp2, eIF4G, and Pab1 under normal growth conditions, yet *ENO2* mRNA does not accumulate to these granules. So deletion of *TPK3* appears to give rise to aberrant granules under normal growth conditions [19]. Further characterization of *tpk3*Δ mutant cells reveals that these aberrant granules contain Pbp1 and Rpg1. As noted previously [19], even though these aberrant granules are present in the cells, the overall polysome profile of exponentially growing *tpk3*Δ mutant cells is unaffected. Despite this, the translation factor distribution along the polysome gradient suggests that deletion of *TPK3* promotes a decrease in the level of eIF4G and Pab1 associated with translating ribosomes. Previous results [19] together with those presented here, indicate that the deletion of *TPK3* could lead to the accumulation of protein complexes containing 48S translation factors under normal growth conditions.

After heat stress in the *tpk3*Δ mutant strain, the level of granule-localized Pab1 and Rpg1 translation factors increased, while those of Dcp2 and Pbp1 remained similar to those observed under normal conditions. In addition, for the *tpk3*Δ mutant strain, an impaired global inhibition of translation was evident following severe heat stress, although an analysis of specific mRNAs shows that more subtle translational alterations are apparent. Overall, these results suggest that the Tpk3 catalytic subunit seems to be required for translational inhibition, the accurate translational regulation of mRNA and the formation of mRNP granules both under normal growth conditions and in response to heat stress.

The results presented here demonstrate that Tpk2 and Tpk3 localize differently and their mutants have opposite phenotypes in terms of the translational response to heat stress. Several reports have shown that kinase isoforms can be differentially regulated or possess opposite roles. For instance, Akt isoforms can be differentially regulated after stroke-induced neuronal injury [45] and CK2-dependent phosphorylation contributes to Akt isoform-dependent substrate specificity [46]. Isoforms of GSK3 positively and negatively modulate the expression of IL-12p40 after stimulation of bovine endothelial cells with peptidoglycan [47]. Precedents also exist for PKA in *Candida albicans*, where catalytic PKA isoforms have opposing roles in glycogen metabolism [48] and in *S*. *cerevisiae* various regulatory mechanisms are targeted to particular Tpk isoforms. So while the different *S*. *cerevisiae* PKA isoforms can have similar substrate specificity *in vitro* [49], several studies suggest that they can have different roles *in vivo*. For example, Tpk1 and Tpk2 differentially control iron metabolism [35, 50]. The three catalytic subunits of PKA play distinct roles in filamentous growth, with Tpk2 serving as an activator and Tpk1 and Tpk3 functioning as inhibitors [51]. Furthermore, a negative isoform-dependent autoregulatory mechanism has been described that controls the expression of *TPK1*, *TPK2*, *TPK3* and *BCY1* [52]. In terms of their physical interactions, data available on the Saccharomyces Genome Database [53] indicates that Tpk1, Tpk2 and Tpk3 physically interact with 320, 98 and 114 proteins, respectively, while 87.6% of these proteins interact with only one of the Tpks. In terms of the varying isoform-specific functions of PKA, the relative localization of the catalytic subunits appears to play an important role: distinct nuclear-cytoplasmic localization or PB/SG localizations have been observed for each Tpk in response to different stimuli such as nutrient availability and hyperosmotic stress [18, 19, 28]. Also, differential phosphorylation status of each Tpk could contribute to divergence in their catalytic activity and cAMP-dependent regulation [25, 54].

Overall, our results show that PKA affects the translational response to heat stress, where each Tpk catalytic isoform appears to have a different role, with Tpk2 and Tpk3 playing negative and positive roles in the translation response, respectively. The mechanism would not appear to be the inactivation of enzymatic activity of Tpk2/3 since its intrinsic catalytic activity is conserved after severe heat stress. Moreover, active Tpk2 molecules are necessary for their location in SG. We favor a model where depending on the severity of an external stimulus, such as heat stress, each catalytic isoform of PKA interacts with a complex network of distinct protein factors and potential substrates. Furthermore, the isoforms may themselves be differentially modified as a consequence of some of these interactions. Therefore, our developing studies are focused on a global characterization of Tpk-associated protein complexes under different conditions.

## Supporting information

S1 Fig Protein expression levels and biochemical analysis on granule-enriched fractions experiments. (A) Expression levels of each PKA subunit under exponential growth (30°C) or after heat stress conditions as described in Fig 1A (37°C or 46°C) were determined by immunoblot with anti-GFP. (B) Expression levels of translation factors in WT, *tpk2*Δ and *tpk3*Δ analysis, western blot from protein extracts obtained from exponentially growth cells (30°C) or mild heat stressed (37°C for 30 minutes) and severe heat stressed (46°C for 10 min). The numbers under the blots represent the densitometric quantification relative to the Pyk1 bands. (C) Soluble and granular fractions from a WT strain after severe heat stress with or without cycloheximide (+CHX or -CHX) treatment before heat stress. The graph shows the ratio P/S of the abundance of each protein determined by densitometric quantification of the bands. (D) Polysomal profile analysis and immunoblots of 15–50% sucrose gradient fractions from *tpk1*Δ cells grown to exponential phase in YPD (30°C) and subjected to severe heat stress (46°C for 10 minutes). The numbers represent the polysome/monosome area ratio. (E) The graph shows the % of each translation factor in monosome/free fraction (M/F) and polysome fraction (P) determined by densitometric quantification of the bands. (F) Pab1-GFP granule size (μm^2^) of wild type and *tpk2*Δ cells after severe heat stress was measured by manual particle analysis using Image J (National Institutes of Health) for at least 40 cells. The value represents mean +/- SEM, n = 40. *p < 0.05 (Man-Whitney T test). (G) Upper panel. Protein kinase activity was assayed in equivalent aliquots of the purified GFP-Tpk1-His_6_, GFP-Tpk2-His_6_ or GFP-Tpk3-His_6_ samples isolated from wild type cells pre and post heat stress 46°C 10 minutes. PKA specific activity of each Tpk was calculated as the total catalytic activity compared with the amount of purified Tpk-tagged protein quantified by densitometric analysis from equivalent samples subjected to SDS/PAGE followed by immunoblotting with anti-His (lower image, an asterisk denotes unspecific bands). Total catalytic activity is expressed as U (units) in each sample defined as pmol kemptide phosphorylated. min^-1^ under the standard conditions. The value represents mean +/- SEM, n = 2.
(TIF)

S2 Fig Cell viability assay. Strains were grown to exponential phase at 30°C and subjected to 37°C 30 minutes, 46°C 10 minutes or 46° C 30 minutes. Cell viability was verified by spot assay.
(TIF)

S1 Tables Table A. Strains used in this study. Table B. Plasmids used in this study. Table C. Primers used in this study.
(DOCX)
