# Supplementary figures and images for: The role of PKA in the translational response to heat stress in Saccharomyces cerevisiae
Source: PLoS One. 2017 Oct 18;12(10):e0185416. doi: 10.1371/journal.pone.0185416 (PMC5646765; doi:10.1371/journal.pone.0185416)

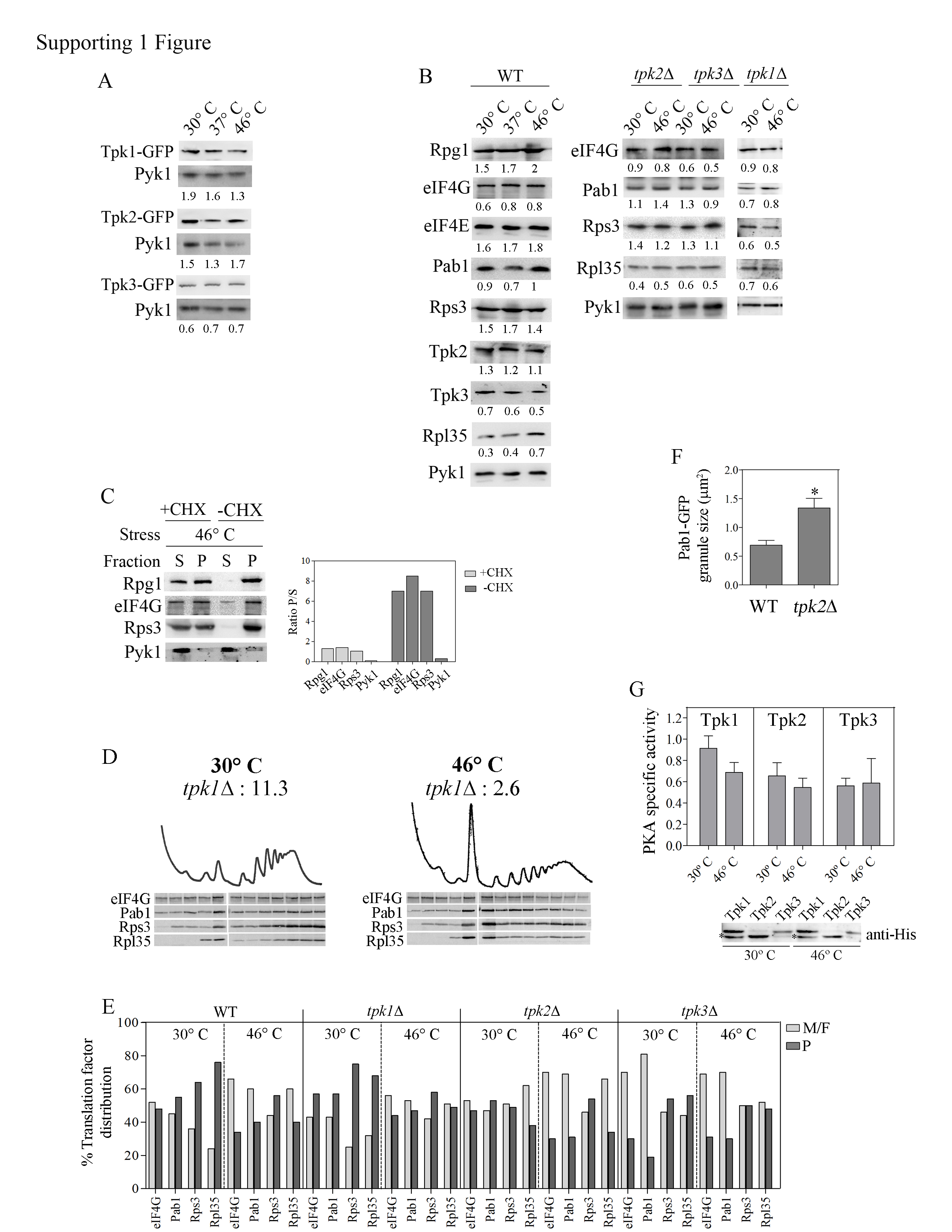

Supplement: S1 Fig — (A) Expression levels of each PKA subunit under exponential growth (30°C) or after heat stress conditions as described in Fig 1A (37°C or 46°C) were determined by immunoblot with anti-GFP. (B) Expression levels of translation factors in WT, tpk2Δ and tpk3Δ analysis, western blot from protein extracts obtained from exponentially growth cells (30°C) or mild heat stressed (37°C for 30 minutes) and severe heat stressed (46°C for 10 min). The numbers under the blots represent the densitometric quantification relative to the Pyk1 bands. (C) Soluble and granular fractions from a WT strain after severe heat stress with or without cycloheximide (+CHX or -CHX) treatment before heat stress. The graph shows the ratio P/S of the abundance of each protein determined by densitometric quantification of the bands. (D) Polysomal profile analysis and immunoblots of 15–50% sucrose gradient fractions from tpk1Δ cells grown to exponential phase in YPD (30°C) and subjected to severe heat stress (46°C for 10 minutes). The numbers represent the polysome/monosome area ratio. (E) The graph shows the % of each translation factor in monosome/free fraction (M/F) and polysome fraction (P) determined by densitometric quantification of the bands. (F) Pab1-GFP granule size (μm2) of wild type and tpk2Δ cells after severe heat stress was measured by manual particle analysis using Image J (National Institutes of Health) for at least 40 cells. The value represents mean +/- SEM, n = 40. *p < 0.05 (Man-Whitney T test). (G) Upper panel. Protein kinase activity was assayed in equivalent aliquots of the purified GFP-Tpk1-His6, GFP-Tpk2-His6 or GFP-Tpk3-His6 samples isolated from wild type cells pre and post heat stress 46°C 10 minutes. PKA specific activity of each Tpk was calculated as the total catalytic activity compared with the amount of purified Tpk-tagged protein quantified by densitometric analysis from equivalent samples subjected to SDS/PAGE followed by immunoblotting with anti-His (lower image, an [file pone.0185416.s001.tif]

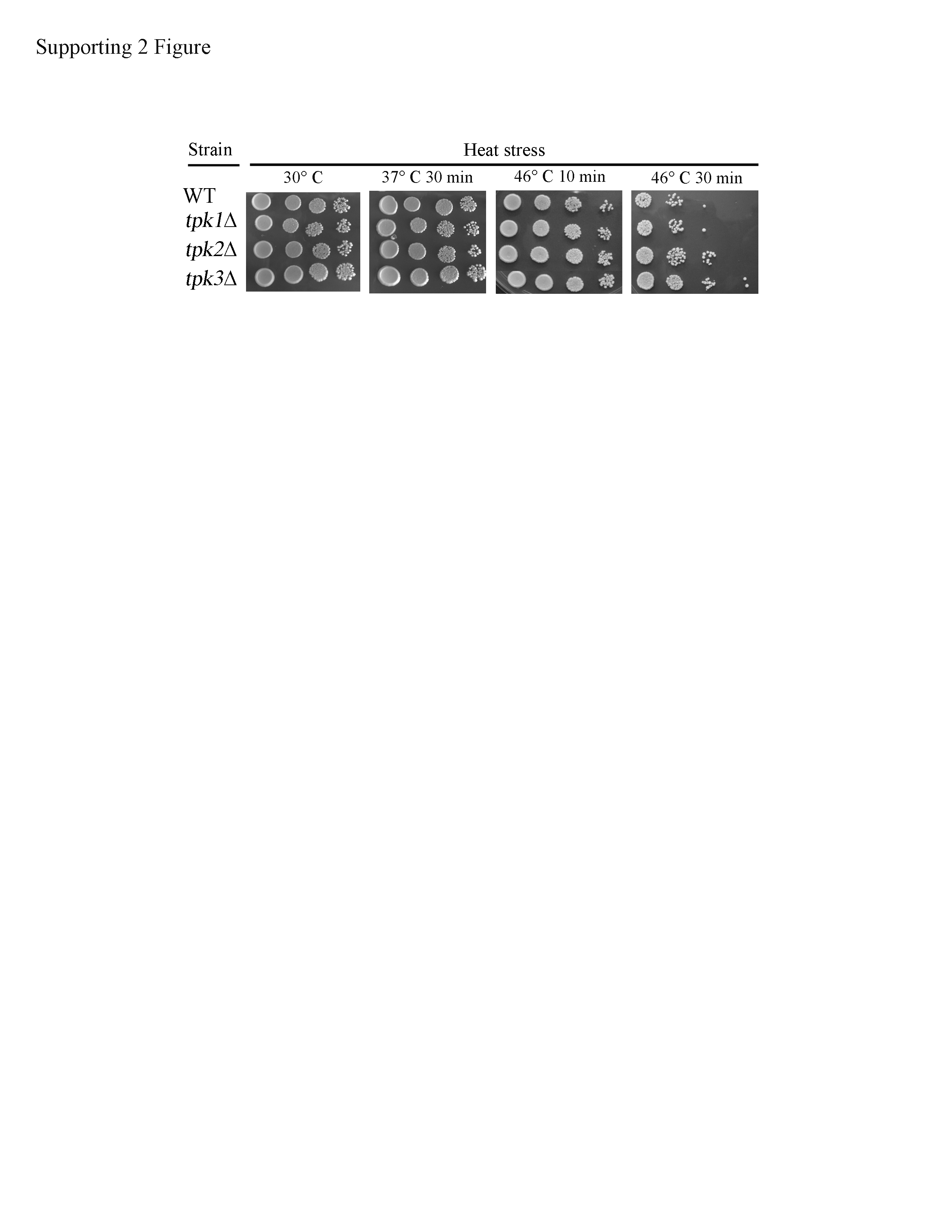

Supplement: S2 Fig — Strains were grown to exponential phase at 30°C and subjected to 37°C 30 minutes, 46°C 10 minutes or 46° C 30 minutes. Cell viability was verified by spot assay. (TIF) [file pone.0185416.s002.tif]
