# Supplementary material for: The role of PKA in the translational response to heat stress in Saccharomyces cerevisiae
Source: PLoS One. 2017 Oct 18;12(10):e0185416. doi: 10.1371/journal.pone.0185416 (PMC5646765; doi:10.1371/journal.pone.0185416)
Supplement: S1 Tables — Table A. Strains used in this study. Table B. Plasmids used in this study. Table C. Primers used in this study. (DOCX) [file pone.0185416.s003.docx]

**S1 Tables**.

Table A. Strains used in this study.

| **Strain** | **Genotype** | **Source** |
| --- | --- | --- |
| W303-1A (WT) | *MATa; ade2-1; his3-11, 15; leu2-3, 112; trp1Δ2; ura3-1; can1-100* | EUROSCARF |
| S322 *(∆tpk1)* | *MATa ade2-1 trp1-1 can1-100 leu2-3,112 his3-11,15 ura3 GAL psi+ tpk1 : : Kan-R* | *[*[*1*](#_ENREF_1)*]* |
| S323 *(∆tpk2)* | *MATa ade2-1 trp1-1 can1-100 leu2-3,112 his3-11,15 ura3 GAL psi+ tpk2 : : Kan-R* | *[*[*1*](#_ENREF_1)*]* |
| S327 *(∆tpk3)* | *MATa ade2-1 trp1-1 can1-100 leu2-3,112 his3-11,15 ura3 GAL psi+ tpk3 : : Kan-R* | *[*[*1*](#_ENREF_1)*]* |
| *BCY1-GFP* | *MATa leu2Δ0 met15Δ0 ura3Δ0 his3Δ1 BCY1-GFP:: HIS3MX* | *Invitrogen* |
| *TPK1-GFP* | *MATa leu2Δ0 met15Δ0 ura3Δ0 his3Δ1 TPK1-GFP:: HIS3MX* | *Invitrogen* |
| *TPK2-GFP* | *MATa leu2Δ0 met15Δ0 ura3Δ0 his3Δ1 TPK2-GFP:: HIS3MX* | *Invitrogen* |
| *TPK3-GFP* | *MATa leu2Δ0 met15Δ0 ura3Δ0 his3Δ1 TPK3-GFP:: HIS3MX* | *Invitrogen* |
| *RPG1-RFP*  *TPK2-GFP* | *MATa leu2Δ0 met15Δ0 ura3Δ0 his3Δ1 RPG1-RFP::NAT TPK2-GFP:: HIS3* | *This study* |
| *RPG1-RFP*  *TPK3-GFP* | *MATa leu2Δ0 met15Δ0 ura3Δ0 his3Δ1 RPG1-RFP::NAT TPK3-GFP:: HIS3* | *This study* |
| *eIF4E-RFP*  *TPK2-GFP* | *MATα ADE2,15 leu 2-3,112 trp 1-1 ura3-1 CDC33-RFP::NAT TPK2-GFP::HIS* | *[*[*2*](#_ENREF_2)*]* |
| *eIF4E-RFP*  *TPK3-GFP* | *MATα ADE2,15 leu 2-3,112 trp 1-1 ura3-1 CDC33-RFP::NAT TPK3-GFP::HIS* | *[*[*2*](#_ENREF_2)*]* |
| *DCP2-RFP*  *TPK2-GFP* | *MATa ade2-1 trp1-1 can1-100 leu2-3,112 his3-11,15 ura3 GAL psi+ tpk2::Kan-R [pDCP2-RFP] [pTPK2-GFP]* | *[*[*2*](#_ENREF_2)*]* |
| *DCP2-RFP*  *tpk2^dead^-GFP* | *MATa ade2-1 trp1-1 can1-100 leu2-3,112 his3-11,15 ura3 GAL psi+ tpk2::Kan-R [pDCP2-RFP] [ptpk2dead-GFP]* | *[*[*2*](#_ENREF_2)*]* |
| *DCP2-RFP*  *TPK3-GFP* | *MATa ade2-1 trp1-1 can1-100 leu2-3,112 his3-11,15 ura3 GAL psi+ tpk3::Kan-R [pDCP2-RFP] [pTPK3-GFP]* | *[*[*2*](#_ENREF_2)*]* |
| *DCP2-RFP*  *Tpk3^dead^-GFP* | *MATa ade2-1 trp1-1 can1-100 leu2-3,112 his3-11,15 ura3 GAL psi+ tpk2::Kan-R [pDCP2-RFP] [ptpk3dead-GFP]* | *[*[*2*](#_ENREF_2)*]* |
| *PBP1-GFP*  *EDC3-RFP* | *MATa; ade2-1; his3-11, 15; leu2-3, 112; trp1Δ2; ura3-1; can1-100 [pPBP1-GFP,EDC3-mCherry]* | *This study* |
| *PBP1-GFP*  *EDC3-RFP ∆tpk1* | *MATa ade2-1 trp1-1 can1-100 leu2-3,112 his3-11,15 ura3 GAL psi+ tpk1::Kan-R [pPBP1-GFP,EDC3-mCherry]* | *This study* |
| *PBP1-GFP*  *EDC3-RFP ∆tpk2* | *MATa ade2-1 trp1-1 can1-100 leu2-3,112 his3-11,15 ura3 GAL psi+ tpk2::Kan-R [pPBP1-GFP,EDC3-mCherry]* | *This study* |
| *PBP1-GFP*  *EDC3-RFP ∆tpk3* | *MATa ade2-1 trp1-1 can1-100 leu2-3,112 his3-11,15 ura3 GAL psi+ tpk3::Kan-R [pPBP1-GFP,EDC3-mCherry]* | *This study* |
| *DCP2-CFP eIF4E-RFP*  *∆tpk3*  *TPK2-GFP* | *Matα ADE2 his3-11,15 leu2-3 112 trp1-1 ura 3-1 can1-100 GCD1-S180 Dcp2-CFP-Trp CDC33-RFP-Nat tpk3::URA3 [pTPK2-GFP]* | *This study* |
| *Rpg1-RFP Dcp2-GFP* | *MATa leu2-3112 ura3-52 his3-Δ200 RPG1::RFP::KanMX DCP2::GFP::HIS3MX* | *[*[*3*](#_ENREF_3)*]* |
| *Rpg1-RFP Pab1-GFP* | *MATa leu2-3112 ura3-52 his3-Δ200 RPG1::RFP::KanMX PAB1::GFP::HIS3MX* | *[*[*3*](#_ENREF_3)*]* |
| *Rpg1-RFP Dcp2-GFP ∆tpk1* | *MATa leu2-3112 ura3-52 his3-Δ200 RPG1::RFP::KanMX DCP2::GFP::HIS3MX tpk1::URA3* | *This study* |
| *Rpg1-RFP Pab1-GFP ∆tpk1* | *MATa leu2-3112 ura3-52 his3-Δ200 RPG1::RFP::KanMX PAB1::GFP::HIS3MX tpk1::URA3* | *This study* |
| *Rpg1-RFP Dcp2-GFP ∆tpk2* | *MATa leu2-3112 ura3-52 his3-Δ200* *RPG1::RFP::KanMX DCP2::GFP::HIS3MX tpk2::URA3* | *This study* |
| *Rpg1-RFP Pab1-GFP ∆tpk2* | *MATa leu2-3112 ura3-52 his3-Δ200 RPG1::RFP::KanMX PAB1::GFP::HIS3MX tpk2::URA3* | *This study* |
| *Rpg1-RFP Dcp2-GFP ∆tpk3* | *MATa leu2-3112 ura3-52 his3-Δ200 RPG1::RFP::KanMX DCP2::GFP::HIS3MX tpk3::URA3* | *This study* |
| *Rpg1-RFP Pab1-GFP ∆tpk3* | *MATa leu2-3112 ura3-52 his3-Δ200 RPG1::RFP::KanMX PAB1::GFP::HIS3MX tpk3::URA3* | *This study* |

Table B. Plasmids used in this study.

| **Plasmid** | **Description** | **Source** |
| --- | --- | --- |
| *pTPK2-GFP* | *pTD46, CEN, LEU2, TPK2 promotor, TPK2-GFP::HIS3* | *[*[*2*](#_ENREF_2)*]* |
| *ptpk2dead-GFP* | *pTD55, CEN, LEU2, TPK2 promoter, tpk2K99M-GFP::HIS3* | *[*[*2*](#_ENREF_2)*]* |
| *pTPK3-GFP* | *pTD49, CEN, URA3, TPK3 promotor, TPK3-GFP::HIS3* | *[*[*2*](#_ENREF_2)*]* |
| *ptpk3dead-GFP* | *pTD61, CEN, URA3, TPK3 promoter, tpk3K117R-GFP::HIS3* | *[*[*2*](#_ENREF_2)*]* |
| *pDCP2-RFP* | *pRP1186, CEN, TRP1, DCP2 promotor, DCP2-RFP* | *[*[*4*](#_ENREF_4)*]* |
| *pPBP1-GFP,EDC3-mCherry* | *pRP1944, CEN, TRP1, PBP1 promotor, PBP1-GFP, EDC3 promotor, EDC3-mCherry* | *[*[*5*](#_ENREF_5)*]* |
| *pRFP::NAT* | *pYM42, RedStar::natNT2* | *[*[*6*](#_ENREF_6)*]* |
| *pRS406* | *pRS406, CEN, URA3* | *Portela lab.* |
| pBEVYU-GFP-Tpk1-His_6_ | *URA3, ADH1 promotor, GFP-TPK1-His_6_* | *[*[*7*](#_ENREF_7)*]* |
| pBEVYU-GFP-Tpk2-His_6_ | *URA3, ADH1 promotor, GFP-TPK2-His_6_* | *[*[*7*](#_ENREF_7)*]* |
| pBEVYU-GFP-Tpk3-His_6_ | *URA3, ADH1 promotor, GFP-TPK3-His_6_* | *[*[*7*](#_ENREF_7)*]* |

Table C. Primers used in this study.

| **Primer** | **Sequence** |
| --- | --- |
| Rpg1-RFP F | TGACTATGGCTGAAAAGTTGAGAGCCAAGAGATTGGCCAAGGGGGGCAGGCAGGTCGACGGAGCTGGAGC |
| Rpg1-RFP R | CTTATACGTATAAAACGGTATAAATTAAGTAGATCATTTTGCGTTGTCTGGCTCGATTACAACAGGTGTTG |
| Tif32 F | CGTGCTCAAGAACTCGCTGA |
| Nat1158 R | GGTACCCATGGTTGTTTATG |
| Tpk1-URA For | AGTGCATGAATTATAGCTGATTGTGTGAAAGAATCTTTTTTTTGGGTATGGCAGATTGTACTGAGAGTGC |
| Tpk1-URA Rev | ATGAAAAAAAAAAAATATAGATACGAGAGGAAAATACAACAAAACATTAGTCACTGTGCGGTATTTCACACCG |
| Tpk2-URA For | CAGCAGCTTCACTCAGGTTAACTCACATACTGTTGAAAATTGTCGGTATGGCAGATT GTACTGAGAGTGC |
| Tpk2-URA Rev | AGAGAAAGTACTTGAAAATTGTTTTTGTGTTTTTTGGTTCATGGAACTTACTGTGCGGTATTTCACACCG |
| Tpk3-URA For | ATATTGTATATCGGTGGTTGTACAAGGAAAGAGCGAGCCTGCACAAAATGGCAGATTGTACTGAGAGTGC |
| Tpk3-URA Rev | CTTTATTGATTTTTTTTTTTTTTCAATTACAATTATCCCACTGAACCTCCCTGTGCGGTATTTCACACCG. |
| Tpk1 VTF | ATCTTGAATGCCGAATTGAG |
| Tpk2 VF | GAGAAGACAGCATCAATTCG |
| Tpk3 VF | TCGGTAAGAAACTCGAGTGG |
| URA3AS2R | CTGGCCGCATCTTCTCAAATATGC |
| Luc For | ACGTCTTCCCGACGATGA |
| Luc Rev | GTCTTTCCGTGCTCCAAAAC |
| ENO2 F | TGACTTGACTGTCACCAACCCAGC |
| ENO2 R | GGAAGTTTTCACCGGCGTAG |
| HSP42 a F | CGGGCATCATCCTCGTCATCATC |
| HSP42 a R | tcttctcgagttgctgtcctc |
| HSP30 F | GTCTAAGTGATGGTGGTAAC |
| HSP30 R | CTAAGCAGTATCTTCGACAG |
| CYC1 F | AAACTTGCATGGTATCTTTG |
| CYC1 R | AGGTAATTAAGTCGTTTCTGT |

References.

1. Bolte M, Dieckhoff P, Krause C, Braus GH, Irniger S. Synergistic inhibition of APC/C by glucose and activated Ras proteins can be mediated by each of the Tpk1-3 proteins in Saccharomyces cerevisiae. Microbiology. 2003;149(Pt 5):1205-16. doi: 10.1099/mic.0.26062-0. PubMed PMID: 12724382.

2. Tudisca V, Simpson C, Castelli L, Lui J, Hoyle N, Moreno S, et al. PKA isoforms coordinate mRNA fate during nutrient starvation. J Cell Sci. 2012;125(Pt 21):5221-32. doi: 10.1242/jcs.111534. PubMed PMID: 22899713; PubMed Central PMCID: PMCPMC3533396.

3. Grousl T, Ivanov P, Frydlova I, Vasicova P, Janda F, Vojtova J, et al. Robust heat shock induces eIF2alpha-phosphorylation-independent assembly of stress granules containing eIF3 and 40S ribosomal subunits in budding yeast, Saccharomyces cerevisiae. J Cell Sci. 2009;122(Pt 12):2078-88. doi: 10.1242/jcs.045104. PubMed PMID: 19470581.

4. Teixeira D, Sheth U, Valencia-Sanchez MA, Brengues M, Parker R. Processing bodies require RNA for assembly and contain nontranslating mRNAs. RNA. 2005;11(4):371-82. doi: 10.1261/rna.7258505. PubMed PMID: 15703442; PubMed Central PMCID: PMCPMC1370727.

5. Swisher KD, Parker R. Localization to, and effects of Pbp1, Pbp4, Lsm12, Dhh1, and Pab1 on stress granules in Saccharomyces cerevisiae. PloS one. 2010;5(4):e10006. doi: 10.1371/journal.pone.0010006. PubMed PMID: 20368989; PubMed Central PMCID: PMC2848848.

6. Janke C, Magiera MM, Rathfelder N, Taxis C, Reber S, Maekawa H, et al. A versatile toolbox for PCR-based tagging of yeast genes: new fluorescent proteins, more markers and promoter substitution cassettes. Yeast. 2004;21(11):947-62. doi: 10.1002/yea.1142. PubMed PMID: 15334558.

7. Haesendonckx S, Tudisca V, Voordeckers K, Moreno S, Thevelein JM, Portela P. The activation loop of PKA catalytic isoforms is differentially phosphorylated by Pkh protein kinases in Saccharomyces cerevisiae. Biochem J. 2012;448(3):307-20. doi: 10.1042/BJ20121061. PubMed PMID: 22957732.
